# Useable diffraction data from a multiple microdomain-containing crystal of *Ascaris suum* As-p18 fatty-acid-binding protein using a microfocus beamline

**DOI:** 10.1107/S1744309112026553

**Published:** 2012-07-31

**Authors:** Mads Gabrielsen, Alan Riboldi-Tunnicliffe, Marina Ibáñez-Shimabukuro, Kate Griffiths, Andrew J. Roe, Alan Cooper, Brian O. Smith, Betina Córsico, Malcolm W. Kennedy

**Affiliations:** a Institute of Infection, Immunity and Inflammation, University of Glasgow, Glasgow, Scotland; b Australian Synchrotron, Clayton, Victoria, Australia; c Instituto de Investigaciones Bioquimicas de La Plata, CONICET–UNLP, Facultad de Ciencias Medicas, Universidad Nacional de La Plata, La Plata, Argentina; d Institute of Biodiversity, University of Glasgow, Glasgow, Scotland; e School of Chemistry, University of Glasgow, Glasgow, Scotland; f Institute of Molecular, Cell and Systems Biology, University of Glasgow, Glasgow, Scotland

**Keywords:** fatty-acid-binding proteins, parasitic nematodes, *Ascaris suum*, microfocus beamlines

## Abstract

As-p18 is a fatty-acid-binding protein from the parasitic nematode *Ascaris suum*. Although it exhibits sequence similarity to mammalian intracellular fatty-acid-binding proteins, it contains features that are unique to nematodes. Crystals were obtained, but initial diffraction data analysis revealed that they were composed of a number of ‘microdomains’. Interpretable data could only be collected using a microfocus beamline with a beam size of 12 × 8 µm.

## Abstract 2

As-p18, an unusual fatty-acid-binding protein from a parasitic nematode, was expressed in bacteria, purified and crystallized. The use of a microfocus beamline was essential for data collection.

## 1. Introduction

As-p18 is a developmentally regulated fatty-acid-binding protein that is one of the most abundant components of the perivitelline fluid surrounding the developing larval stages of the parasitic nematode *Ascaris suum* (Mei *et al.*, 1997 ▶). The infective third-stage (L3) larva undergoes developmental arrest until ingestion by the host and may survive for up to seven years in the egg before infection of the host, but little is known of the biochemical and physiological basis of such long-term survival. As-p18 displays sequence similarity to the mammalian group of intracellular fatty-acid-binding proteins (iFABPs), but modelling indicated that there may be differences in its ligand-binding site and the existence of extended loops exposed on the surface of the expected ten-stranded β-barrel (Mei *et al.*, 1997 ▶). These differences could relate to ligand specificity and interaction with as yet uncharacterized protein or membrane interaction partners. Phylogenetic analysis reveals that As-p18, together with other potential homologues and paralogues in *Caenorhabditis elegans*, comprise a distinct protein class (‘nemFABPs’) within the FABP family that is unique to nematodes (Plenefisch *et al.*, 2000 ▶). The presence of a leader peptide in mRNAs encoding As-p18 and direct evidence of its secretion from the synthesizing cell further distinguish this protein, and possibly nemFABPs in general, from FABPs found in other animal groups. The atypical features of As-p18s may reflect adaptation to a specific function within the egg related to the survival of nematode larvae in general, including those of the highly pathogenic agents of filariasis in humans (Kennedy & Harnett, 2001 ▶; Michalski *et al.*, 2002 ▶).

Here, we report crystallization conditions for As-p18 and the utility of microfocus beamlines to analyse its structure. Whilst the crystals showed no obvious signs of disorder under optical microscopy, it became apparent from initial X-ray diffraction observations that the crystals were composed of multiple microdomains. This prevented data collection on a conventional beamline because the diffraction patterns revealed the presence of multiple lattices. Only when the crystals were brought to a microfocus beamline could a complete data set be collected. The data-collection strategy and data processing is presented here.

## 2. Materials and methods

### 2.1. Protein expression and purification

The protein was expressed in *Escherichia coli* containing plasmid pQE-30/As-p18 (Mei *et al.*, 1997 ▶), which encodes the protein with an N-terminal hexahistidine tag (His tag). The plasmid was sequence-verified and transformed into *E. coli* BL21 (DE3) cells harbouring plasmid pREP4, which encodes the *lac* repressor. Cells were grown in LB medium at 310 K, induced with 1.0 m*M* IPTG and harvested 4 h after induction. The cells were lysed by sonication and the protein was purified from the supernatant by Ni–NTA (Novagen, Germany) affinity and size-exclusion (Superdex 75 HR 10/300; GE Healthcare, USA) chromatography.

### 2.2. Crystallization and crystal preparation

The purified As-p18 with the His tag still intact was concentrated to approximately 15 mg ml^−1^, as estimated by absorption at 280 nm using a theoretical extinction coefficient of 28 420 *M* ^−1^ cm^−1^ derived from the aromatic amino-acid composition (Gasteiger *et al.*, 2005 ▶). Crystallization trials were set up using commercially available screens and the sitting-drop vapour-diffusion method. The drops were set up with a 1:1 ratio of protein and reservoir solution and a final volume of 1 µl using a Cartesian nanodrop dispense unit (Digilab, USA). The trays were incubated at 293 K.

**Figure 1:**
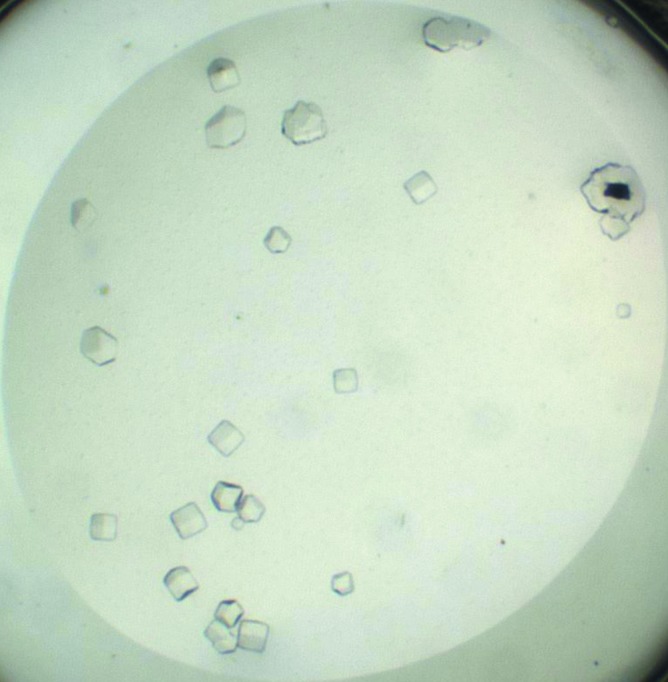
Crystals of As-p18 obtained using 40% ethylene glycol, 0.1 *M* acetate pH 4.5.

Protein crystals appeared within two weeks in condition No. 2 (40% ethylene glycol, 0.1 *M* acetate pH 4.5) of the Cryo I screen (Emerald BioSystems, USA). The largest crystals had maximum dimensions of 0.08 × 0.08 × 0.1 mm (Fig. 1 ▶). Crystals were cooled without any additional cryoprotection in a stream of gaseous nitrogen cooled to 100 K (Oxford Cryosystems, England) and were stored under liquid nitrogen until diffraction testing.

## 3. Results

### 3.1. Data collection

**Figure 2:**
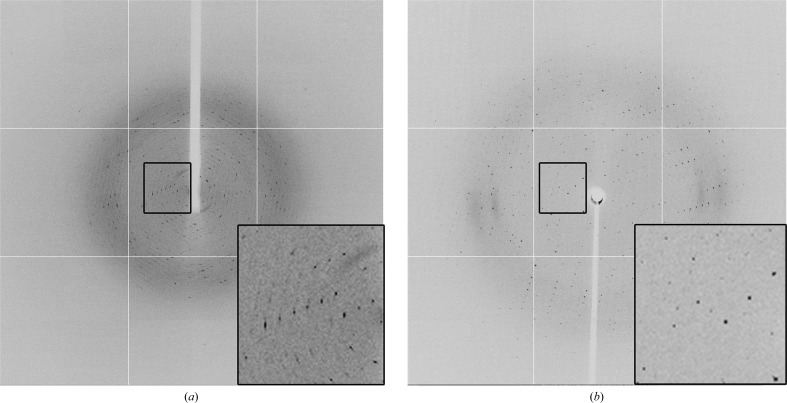
X-ray diffraction images obtained (*a*) at station I04 of DLS using the standard beam size of 71.8 × 75 µm and (*b*) at station MX2 of the Australian Synchrotron using an optimized beam size of 12 × 8 µm.

Crystals were initially tested at station I04 of Diamond Light Source (DLS), UK with an oscillation of 1° at a wavelength of 0.9763 Å. Although externally the crystals appeared to be well formed, the diffraction spots were diffuse and showed signs of aniosotropy (Fig. 2 ▶ *a*). No indexing could be performed to assign the space group.

Using the (then) standard beam size of 71.8 × 75 µm at station I04, diffraction from several of these ‘microdomains’ was collected on each image. A complete processable data set could therefore not be collected on a standard macromolecular crystallography beamline.

Crystals from the same well were transported to station MX2 of the Australian Synchrotron, where using the standard beam size of 35 × 20 µm resulted in similarly unusable diffraction patterns. In order to obtain useful data from these crystals, it was necessary to reduce the beam size to 12 × 8 µm using the microcollimator installed on MX2. Further, owing to the presence of these microdomains within the crystal, it was necessary to screen several (20+) regions of the crystal using the collimated beam.

With a 10 µm aperture, it was possible to scan areas of the crystal to select an area that gave a single lattice in the diffraction pattern (Fig. 2 ▶ *b*). A data set was collected from this region of the crystal, with diffraction being observed to beyond 2.25 Å resolution. Data were collected at a wavelength of 0.9537 Å on an ADSC Quantum 315r detector (crystal-to-detector distance 380 mm) with an oscillation of 0.5° using *Blu-Ice* (McPhillips *et al.*, 2002 ▶).

### 3.2. Data analysis

The crystal belonged to space group *I*4, with unit-cell parameters *a* = *b* = 102.254, *c* = 103.392 Å. Owing to radiation damage, data could be processed to a maximum resolution of 2.3 Å using *XDS* (Kabsch, 2010 ▶) and were reduced using *SCALA* (Evans, 2006 ▶). The data had overall *R*_merge_ and 〈*I*/σ(*I*)〉 values of 8.4% and 6.8, respectively, and values of 49% and 1.4, respectively, in the highest resolution bin. The space group was confirmed by *POINTLESS* (Evans, 2006 ▶) and by manual inspection of the systematic absences. A summary of the data-processing statistics is presented in Table 1 ▶. With a molecular weight of 18 309 Da, the asymmetric unit is most likely to comprise three copies of the protein with a solvent content of 53%. However, two copies and a solvent content of 69% is also possible. The correct composition has yet to be determined.

## 4. Conclusions

We successfully crystallized As-p18 and the crystals diffracted to a resolution of 2.3 Å. Work is currently under way to solve the structure using molecular-replacement methods based on the structures of mammalian FABPs in the Protein Data Bank (PDB). The only way that a quality diffraction pattern could be achieved was by using a microfocus beamline, and this paper highlights the usefulness of microfocus beamlines even for crystals that are apparently large enough to be analysed using regular beamlines.

## Figures and Tables

**Table 1:** Data-collection and reduction statistics Values in parentheses are for the highest resolution bin.

Space group	*I*4
Unit-cell parameters (Å)	*a* = 102.635, *c* = 103.392
Resolution (Å)	20.06–2.30 (2.42–2.30)
Observed reflections	80135
Unique reflections	21931
Multiplicity	3.7 (1.9)
Completeness (%)	93.2 (77.1)
Matthews coefficient (Å^3^ Da^−1^)	3.97/2.65†
Solvent content (%)	69.03/53.55†
Monomers in asymmetric unit	2/3†
*R*_meas_ (%)	10.4 (67.9)
*R*_p.i.m._ (%)	4.9 (43.5)
〈*I*/σ(*I*)〉	9.8 (1.8)
Wilson *B* (Å^2^)	41.7

† The nature of the asymmetric unit has not yet been clarified, but it is expected to contain two or three subunits.
